# APOBEC3B regulates HPV replication by inducing R-loop formation and DNA damage

**DOI:** 10.1371/journal.ppat.1014088

**Published:** 2026-03-23

**Authors:** Conor W. Templeton, Jasmine S. Gulik, Laimonis A. Laimins

**Affiliations:** Microbiology-Immunology Department, Feinberg School of Medicine, Northwestern University, Chicago, Illinois, United States of America; University of North Carolina at Chapel Hill, UNITED STATES OF AMERICA

## Abstract

APOBECs are cytidine deaminases whose levels are increased in cells with high-risk HPV genomes and are responsible for most mutations in HPV associated cancers. APOBEC3B is a nuclear member of this family and is shown to be a positive regulator of HPV replication as well as expression. The proviral effects of A3B found in HPV positive cells contrast with its role as a restriction factor for many other viruses. Studies demonstrated that A3B can bind and regulate the formation of R-loops, which are trimeric nucleic acid structures consisting of an RNA paired with its complementary DNA strand, displacing one of the DNA strands. The present study demonstrates that A3B binds stably to both cellular and viral chromatin at sequences containing high R-loop levels, including the HPV URR and early polyadenylation sites. Importantly, A3B was found to play a critical role in the replication of HPV genomes and in regulating viral expression. Reduction of R-loop levels through overexpression of the R-loop specific RNase, RNase H1, impaired A3B binding to viral genomes as well as at multiple cellular sites. When A3B was depleted, total R-loop levels decreased by ~50%, leading to impaired viral transcription and an increase in the expression of immune genes, such as OASL, IL6, and IRF1. Mapping R-loop formation in A3B depleted cells revealed that A3B regulated a subset of R-loops that form on the transcriptional start (TSS) and termination sites (TTS) of cellular genes, including at the HPV URR. Furthermore, A3B depletion resulted in over a 50% reduction of DNA breaks along with altered expression of DNA damage repair proteins. This study demonstrates that A3B is an inducer of R-loop formation and DNA damage in HPV positive cells, thereby regulating cellular and viral gene expression along with HPV replication.

## Author summary

The APOBEC3 (A3) family of proteins plays a significant role in the generation of mutations in the development of HPV positive cancers. A3 levels are increased in HPV positive cancers that often contain only integrated copies of the HPV genome, as well as in precancerous lesions that contain viral episomes, but little is known about their role in viral pathogenesis. In this study, A3B, one of the cytosine deaminases within the A3 family, was found to be present at high levels in cells that maintain viral episomes, and these elevated levels were shown to be crucial for viral DNA maintenance and gene expression. This is in contrast to A3B’s role as a restriction factor during the life cycle of other DNA viruses. Increased A3B expression in HPV positive cells induced R-loop formation as well as DNA damage, which was important for regulating the expression of a subset of cellular genes. In addition, many, but not all, of the R-loops within HPV positive cells are bound by A3B, including those on the viral URR and early polyA site. Knockdown of A3B reduced R-loop formation at the URR, and similar effects were observed on cellular genes, altering their expression. These data indicate that A3B acts as a proviral factor in HPV replication by regulating R-loop formation and cellular and viral gene expression.

## Introduction

Human papillomaviruses (HPVs) are the causative agents of over 5% of all human cancers, including squamous cell carcinomas of the cervix and oropharynx [1–3]. Of the over 400 types of HPVs identified, 15 have been associated with the development of cancer and are designated as high-risk [4]. High-risk HPVs belong to the alpha papillomaviridae family and include HPV-16, 18, and 31, among others [5]. HPVs infect keratinocytes in the basal layer of epithelia, where they establish their genomes as extrachromosomal episomes at copy numbers of approximately 50–100 [6–8]. The production of virions, however, requires differentiation and is restricted to suprabasal layers [9]. The maintenance of viral genomes, along with productive replication, requires the activation of the ataxia telangiectasia mutated (ATM) and ataxia telangiectasia mutated and rad3-related (ATR) DNA repair pathways [10,11]. Both these pathways are activated through the induction of high levels of DNA breaks caused by the major viral oncoproteins, E6 and E7 [12–14]. E7 induces DNA breaks through increased expression of topoisomerases, while E6 acts by increasing the formation of R-loops, which are trimeric RNA:DNA hybrids consisting of RNA stably paired with its complementary DNA, along with the opposite displaced DNA strand [12,13,15]. R-loops form at promoter and terminator regions to regulate expression and are highly enriched in HPV positive cells [16–18], while aberrant R-loop resolution or formation results in the accumulation of DNA breaks [19–21].

HPV positive lesions can persist for extended periods, as viral genomes are tethered to cellular chromatin, allowing for similar copy numbers to be maintained through cell division [22–24]. Persistent high-risk HPV infections can, however, progress to cancers, and these are often associated with integration of viral genomes into host chromatin [25,26]. Integration restricts viral gene expression to the two major oncoproteins, E6 and E7 [27]. Expression of the E6 and E7 oncoproteins alone is not sufficient to induce cancers but also requires the acquisition of numerous mutations in the cellular genome [28,29]. The primary drivers of these mutations in HPV positive cells are the apolipoprotein B mRNA-editing enzyme catalytic polypeptide-like 3 (APOBEC3 or A3) family of cytosine deaminases, which act as intrinsic immune sensors [28,30–33]. The levels of two A3 family members, APOBEC3A (A3A) and APOBEC3B (A3B), are elevated in both HPV positive precancerous lesions as well as cancers [34–37]. E6 and E7 have both been shown to increase the expression of A3A and A3B by targeting multiple upstream cellular factors, including TEAD and Rb. The increased expression of A3A and A3B in precancerous HPV positive cells, which stably maintain viral episomes, is surprising, as many other viruses inhibit these factors [38,39]. The replication of viruses like Epstein-Barr virus (EBV), Hepatitis B virus (HBV), and Human Immunodeficiency virus (HIV) can be inhibited by APOBECs, and viral proteins counteract their actions [40–45]. Recently, A3B has been shown to bind to displaced single-stranded DNA (ssDNA) that forms during the formation of R-loops [46]. A3B has been reported to bind to a subset of R-loops and regulate their formation and stability [46,47]. Precancerous cells that stably maintain HPV episomes have been shown to contain increased R-loop levels, which are critical for both viral replication and gene expression [13,16]. The enhanced levels of A3B and R-loops in HPV positive cells suggest a potential linkage that could affect viral replication or expression.

Our studies show that A3B levels are increased in cells that maintain high-risk HPV episomes in comparison to normal cells. Furthermore, A3B was found to bind to the viral genome at the upstream regulatory regions (URR) along with early polyadenylation sites, which are the primary regions where R-loops form. Depletion of A3B using shRNAs impaired viral replication and transcription with minimal effects on cell growth. A3B was also found to induce high levels of DNA breaks and increase global R-loop formation within HPV positive cells, resulting in decreased expression of cellular genes involved in the interferon response, such as IL-6, ISG15, and IFI44. Furthermore, the high levels of A3B in HPV positive cells positively regulated the expression of genes involved in cell proliferation and pol II transcription. A3B was shown to preferentially regulate R-loop formation at promoter and terminator regions in these HPV positive cells. Finally, the reduction of R-loops through the overexpression of the R-loop specific RNase, RNase H1, significantly decreased but did not abrogate A3B binding to both cellular and viral chromatin. These findings indicate that elevated levels of A3B regulate R-loop formation in HPV positive cells, and this is critical for HPV replication.

## Results

### APOBEC3B expression is induced within keratinocytes maintaining HPV episomes

**Fig 1:**
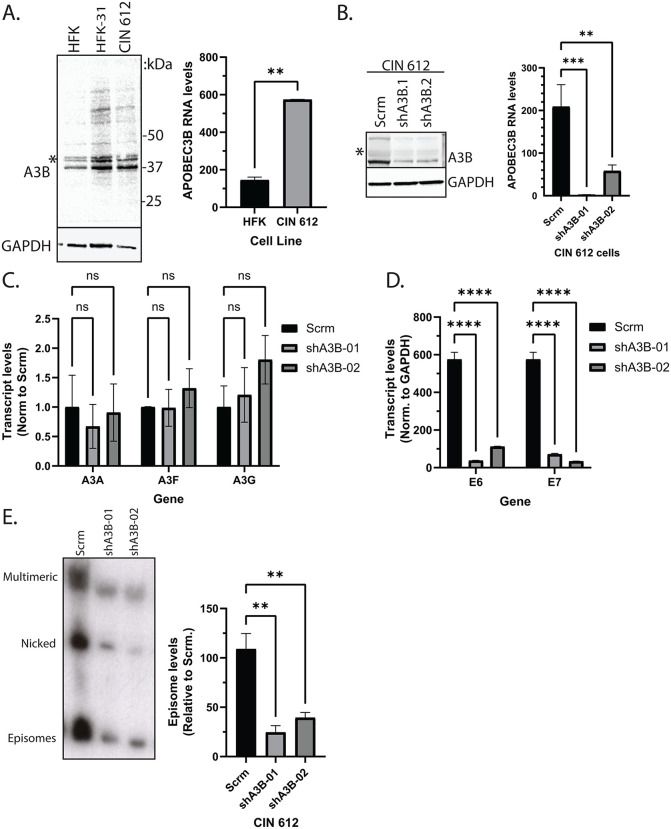
Levels of APOBEC3B are elevated within HPV positive cells and are critical for maintenance of viral genomes and gene expression. (A, left) Western blot analysis of whole cell lysates from HFK, HFK 31, and CIN 612 cells using a previously published A3B antibody [48]. Predicted molecular weight of A3B is ~ 46 kDa, but is observed to resolve ~37 kDa. RT-qPCR analysis of RNA extracts from HFK and CIN 612 cells (A, right). (B, left) Western blot analysis of CIN 612 cells depleted of A3B through stable expression of shRNAs. RT-qPCR analysis of RNA extracts from scramble control and two cell lines stably expressing shRNAs targeting A3B (B, right). **(C)** Analysis of expression levels of other A3 family members in scramble control and shA3B CIN 612 cells from total RNA sequencing (n = 2, n.s. = not significant). **(D)** RT-qPCR analysis of RNA extracts from scramble control and shA3B CIN 612 cells examining HPV 31 E6 and E7 expression. **(E)** Southern blot analysis of HPV genome levels from scramble control and shA3B CIN 612 cells. Quantification depicted on the right.

The levels of A3B in normal keratinocytes and two cell lines stably maintaining HPV 31 episomes (HFK 31 and CIN 612, respectively) were assessed by western blot analysis using previously verified antibodies [48,49]. Similar to previous reports, A3B resolved at ~37 kDa with a preponderance of higher molecular weight species detected in the HPV positive cell lines, consistent with a pattern of polyubiquitination and other post-translational modifications (Fig 1A, left). A3B RNA levels were also compared between our normal keratinocytes and HPV positive CIN 612 cells, and significant increases were observed in the latter (Fig 1A, right). To validate antibody specificity in our HPV positive cell lines, we generated CIN 612 cells stably expressing shRNAs targeting A3B. Significant reductions in A3B levels were detected, accompanied by similar decreases in the levels of higher molecular weight species (Fig 1B, left). Similar results were observed using a second commercially available antibody (S1B Fig). Additionally, significant reductions in the RNA levels of A3B, but not A3A, A3F, or A3G, were detected (Fig 1B, right and 1C), with minimal effect on cell growth (S1C Fig).

As A3B acts as an antiviral factor during many viral infections, we investigated whether A3B restricted HPV gene expression and genomic maintenance. For this analysis, CIN 612 cells depleted of A3B using shRNAs were examined by RT-qPCR using primers targeting the HPV31 E6 and E7 genes. Depletion of A3B significantly reduced the expression of both E6 and E7 by up to 10-fold compared to the scramble control cells (Fig 1D), and only low levels remained. Next, Southern blot analysis for HPV 31 genomes was performed, revealing a significant reduction in the levels of viral episomes (Fig 1E).

To expand this analysis to other high risk HPV types, A3B expression was examined by western blot analysis within primary keratinocytes stably maintaining HPV 16 and 18 episomes. Similar increases in A3B levels were identified in HFK 16 and 18 cells, (S2A Fig). To test whether A3B acts as a proviral factor during HPV 16 and 18 pathogenesis, A3B was stably depleted using the same shRNA sequences as those used on the CIN 612 cells. Significant reductions in A3B RNA and protein levels were observed, with a corresponding decrease in E6 and E7 expression as soon as one passage post selection (S2B and S2C Fig). These data suggest that A3B acts as a proviral factor during the pathogenesis of multiple high-risk HPVs.

### APOBEC3B interaction with cellular and viral chromatin is promoted by R-loop formation

**Fig 2:**
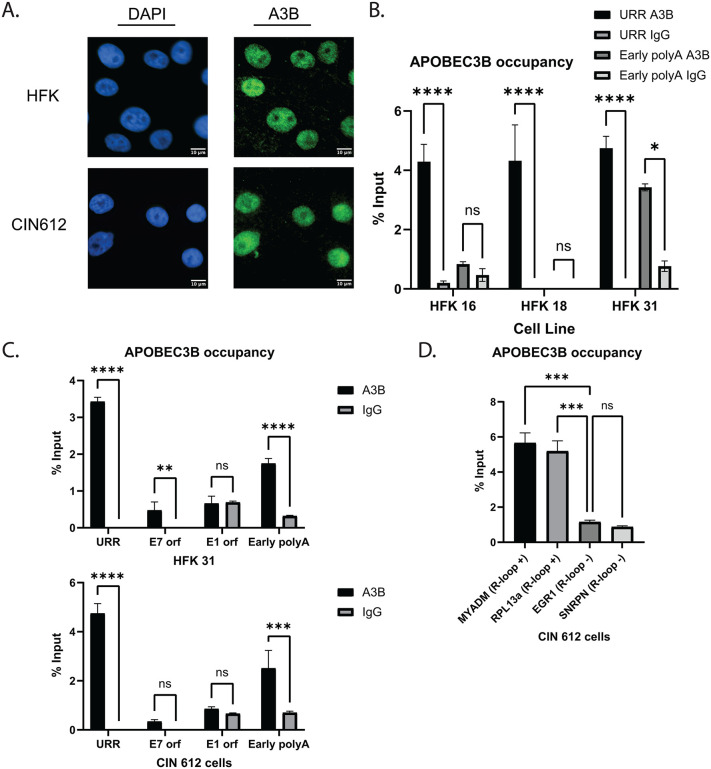
APOBEC3B remains nuclear in HPV positive cells and interacts with viral and cellular chromatin on regions with high R-loop formation. **(A)** Immunofluorescence analysis of HFK and CIN 612 cells using anti-A3B and DAPI. Scale bars = 10μm. **(B)** Chromatin immunoprecipitation-quantitative PCR (ChIP-qPCR) analysis for A3B binding to the HPV URR and early polyA for HPV 16, 18, and 31. The percentage input was plotted: Input% = 100/2^(ΔCt)^ normalized to input controls. **(C)** ChIP-qPCR of HFK 31 and CIN 612 for A3B interaction sites on the HPV 31 genome. The percentage input was plotted. **(D)** ChIP-qPCR of CIN 612 cells for A3B binding to previously identified cellular genes with high R-loop levels in HPV positive cells in comparison to normal (MYADM & RPL13a) and at genes with similar levels of R-loops in HPV positive and normal cells (EGR1 & SNRPN). The percentage input was plotted.

A3B is relocalized during infections with various DNA viruses, including human cytomegalovirus (HCMV), herpes simplex viruses (HSV), Epstein-Barr virus (EBV), and Kaposi’s Sarcoma-associated herpesvirus (KSHV) [50–52]. Since this activity is critical in mitigating the antiviral effects of A3B’s cytosine deaminase activity, we investigated whether A3B localization is affected in HPV positive cells. In HPV positive cells, A3B was found to exhibit a pan-nuclear localization, which was very similar to the distribution seen in normal keratinocytes (Fig 2A). As A3B was found to be primarily nuclear in HPV positive cells, we examined whether it could stably interact with viral episomes. A3B specifically binds to and interacts with trinucleotide repeats, TCA/TCT, resulting in the deamination of the cytosine residue [53,54]. Mapping of TCA and TCT repeats in the HPV 16, 18, and 31 genomes revealed significant numbers [11–20] within the upstream regulator region (URR) of each high-risk type examined (HPV 16, 18 and 31).To investigate if A3B bound to viral DNAs, chromatin immunoprecipitation – quantitative PCR (ChIP-qPCR) was performed using primers that mapped to various regions of the HPV 16, 18, and 31 genomes. A3B was found to be stably associated with HPV 31 genomes at the URR as well as at the early polyA site. Similarly, binding to the URR was detected at the URR regions of cells with HPV 16 and 18 episomes, and less so at the early polyA sites (Fig 2B). A3B binding to the URR and the early polyA site of the HPV 31 genome was detected at significantly higher frequencies as compared to the E1 or E7 ORFs in both HFK 31 and CIN 612 cells (Fig 2C). Notably, the binding of A3B on viral chromatin was detected at regions that were previously associated with R-loop formation. Since recent reports have indicated that A3B interacts with R-loops both *in vitro* and *in vivo*, we assessed A3B binding to four previously characterized cellular loci in HPV 31 positive cells [46,47]. Two of these regions exhibited similar levels of R-loop formation in normal and HPV positive cells (*EGR1* and *SNRPN*), while two others showed elevated R-loop levels (*MYADM* and *RPL13a*) in the latter [16]. ChIP-qPCR assays were used to show increased A3B binding at the two cellular loci containing higher R-loop levels compared to the normal loci (Fig 2D). These data support an association between A3B binding and specific sets of R-loops in HPV positive cells.

**Fig 3:**
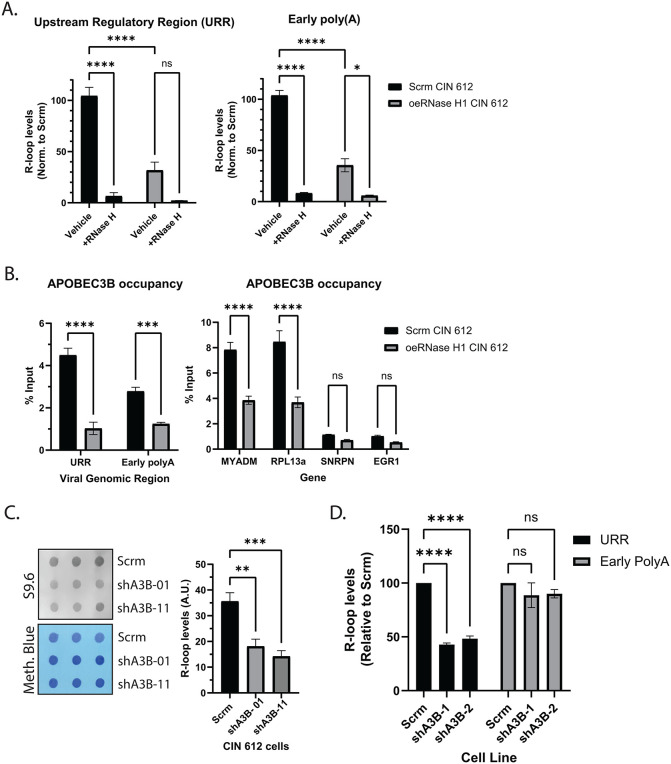
APOBEC3B’s binding to chromatin is facilitated through R-loop formation and is responsible for the induction of~50% of the R-loops present in HPV positive cells. **(A)** DRIP-qPCR analysis of scramble control and RNase H1 overexpressing CIN 612 cells. RNase H treatment was performed post nucleic acid extraction from each condition. R-loop levels were plotted normalized to the scramble control: (S9.6_x_/IgG_x_)/ (S9.6_Scrm_/IgG_Scrm_), where x is Ct values from RNase H treated scramble control, overexpressing RNase H1 vehicle, or RNase H treated. **(B)** APOBEC3B ChIP-qPCR of scramble control and RNase H1 overexpressing CIN 612 cells on R-loop forming regions in viral (left) and cellular (right) chromatin. The percentage input is plotted. **(C)** S9.6 dot blot analysis of nucleic acid extracts from scramble control and A3B depleted CIN 612 cells. Nucleic acid retention on the membrane was normalized to methylene blue staining. **(D)** DRIP-qPCR analysis of R-loop forming regions on the viral chromatin from scramble control and shA3B depleted CIN 612 cells. R-loop levels were plotted normalized to the scramble control: (S9.6_x_/IgG_x_)/ (S9.6_Scrm_/IgG_Scrm_), where x is Ct values from the A3B depleted cell lines.

To assess whether elevated R-loop formation promotes A3B binding to viral and cellular chromatin, HPV positive cells were transduced with lentiviruses to generate cell lines stably expressing the nuclear isoform of RNase H1. RNase H1 is a R-loop specific RNase that resolves these structures by removing the RNA moiety [55,56]. Stable overexpression of RNase H1 in CIN 612 cells was shown to lead to significant reductions in global R-loop levels [13], and we investigated whether this reduction was also observed at several cellular loci. DNA:RNA immunoprecipitations (DRIP) assays using the S9.6 antibody were performed on scramble control and RNase H1-overexpressing CIN 612 cells. Significant reductions in R-loop levels were seen on the viral genome at the URR and early polyA regions when RNase H1 was overexpressed, as well as on cellular loci that exhibited elevated R-loop levels compared to normal keratinocytes (*MYADM* and *RPL13a*) (Figs 3A and S3A). Minimal changes were observed on the loci that contained similar R-loop levels as in normal keratinocytes (*SNRPN* and *EGR1*) (S3B Fig). We next performed ChIP-qPCR to detect A3B binding in scramble control and RNase H1-overexpressing CIN 612 cells, and found significant decreases in A3B association with the viral genome at the URR and early poly(A) site (Fig 3B, left). This trend was also observed in cellular loci that contained elevated R-loop levels in HPV positive cells, such as RPL13a and MYADM (Fig 3B, right), and was not due to reduced transcription of A3B (S3C Fig).

### APOBEC3B promotes R-loop formation in HPV positive cells

Since A3B associates with viral and cellular chromatin at sites of increased R-loop formation, we next asked whether A3B resolves or stabilizes R-loops in HPV positive cells. CIN 612 cells stably expressing shRNAs targeting A3B were first examined using S9.6 dot blot assays to assess effects on global R-loop levels. A3B depletion resulted in a ~ 50% reduction in total R-loop levels (Fig 3C). DRIP-qPCR was then performed using primers targeting the viral genome, and demonstrated reduced R-loop formation at the viral URR when A3B was depleted (Fig 3D). In contrast, no such reduction in R-loops was observed at the early polyA. This suggests that A3B binding influences the formation of R-loops at only a subset of its binding sites, and that other factors may be more dominant than the effects of A3B at these regions.

**Fig 4:**
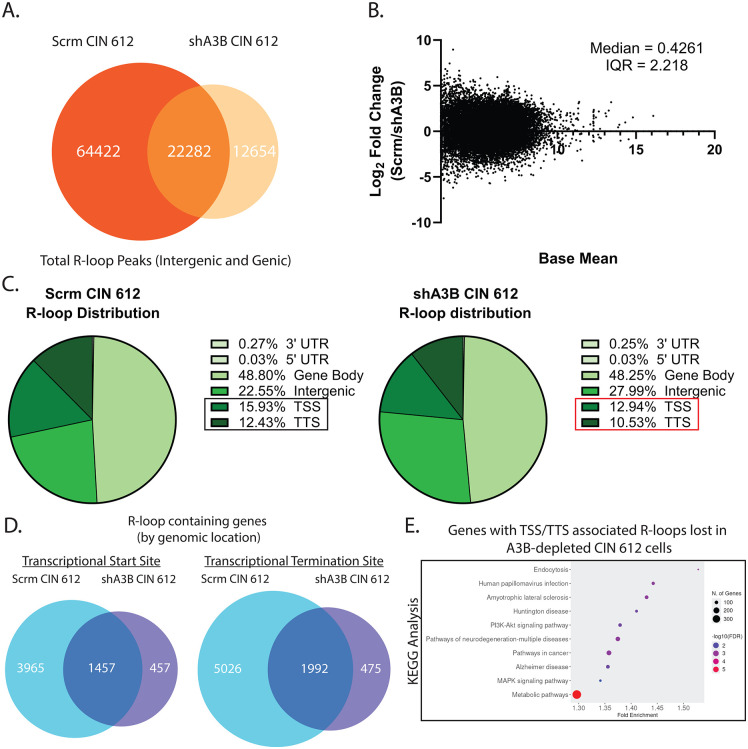
APOBEC3B preferentially regulates R-loop formation at the transcriptional start sites as well as termination sites in HPV positive cells. **(A)** R-loop forming sites identified in the scramble control CIN 612 cells, A3B depleted CIN 612 cells, or both by DRIP-sequencing. **(B)** MA plot analysis of shared R-loop forming sites, where the base mean is equal to the average R-loop reads across all conditions. Median and interquartile range are provided. **(C)** Genomic annotation of R-loop sequences as to whether they form at 3’ UTR, 5’ UTR, gene bodies, intergenic regions, transcriptional start sites, or transcriptional termination sites in scramble control CIN 612 cells (left) or A3B depleted CIN 612 cells (right). **(D)** Venn diagrams depicting genes with R-loops present on their transcriptional start site (left) or termination site (right) in scramble control CIN 612 cells, A3B depleted CIN 612 cells, or both. **(E)** KEGG analysis of genes containing R-loops at their TSS or TTS in scramble control but not A3B depleted CIN 612 cells.

To investigate if A3B binding regulates the formation of R-loops on cellular sequences, DRIP-sequencing was performed on scramble control and A3B-depleted CIN 612 cells. Peak calling identified approximately 87K sites of R-loop formation in the scramble control CIN 612 cells, which is similar to that observed in a previous analysis [16]. In A3B-depleted CIN 612 cells, over 35K sites of R-loop formation were detected, representing a reduction of ~2.5-fold compared to the scramble control (Fig 4A). Analysis of total R-loop forming sequences in A3B-depleted and scramble control cells by MA plot analysis identified higher levels in the scramble control cells compared to A3B depleted cells (Fig 4B), which is consistent with the S9.6 dot blot analyses. These results indicate that A3B depletion reduces, but does not eliminate, R-loop levels in HPV-positive cells.

To understand if R-loop formation at specific sites was affected by A3B binding, sites of R-loop formation from DRIP-sequencing were mapped and annotated for their presence at the 3’ or 5’ UTR of genes, within the gene body itself, at transcriptional start sites (TSS), at transcriptional termination sites (TTS), or in intergenic regions (Fig 4C). In the scramble control cells, over 3/4 of all R-loop peaks occurred near gene bodies with significant enrichments at the TSS (15.93%) and TTS (12.43%), and this is consistent with published DRIP-sequencing analyses [57]. Interestingly, in A3B-depleted CIN 612 cells, a larger proportion of R-loops formed in the intergenic regions (28% vs. 23%), corresponding with a decrease in R-loops forming specifically at TSS and TTS (~3% and 2% reduction, respectively). These data suggest that A3B may preferentially regulate R-loop formation at the TSS and TTS of cellular genes in HPV positive cells.

After annotating the overall genomic locations of R-loop formation dependent upon A3B binding, we next compared the genes containing R-loops that formed at their TSS (Fig 4D, left) or TTS (Fig 4D, right). Scramble control CIN 612 cells contained around 2.5 to 3-fold more genes with R-loops at either TSS or TTS compared to CIN 612 cells depleted of A3B. Additionally, only a small fraction (~7.6% and 6.3%) of genes contained R-loops at TSS or TTS only in the A3B depleted cells. KEGG analysis of the genes containing R-loops at TSS or TTS only in A3B depleted cells did not identify any significantly overrepresented pathways. In contrast, KEGG analysis of the genes associated with R-loops at TSS or TTS in the scramble control cells, but absent in the A3B knockdowns, identified several novel pathways. Among the top pathways identified were those associated with human papillomavirus infection, including DNA damage and interferon-inducible genes, the PI3K-AKT signaling pathway, pathways in cancer, along with metabolic pathways (Fig 4E). When examining the specific genes associated with these overrepresented pathways, factors such as ATR, p53, IFN-α, and EP400 were identified, all of which have known roles in facilitating HPV pathogenesis (S4 Fig) [58–61]. In addition to the top 10 overrepresented pathways displayed in Fig 4E, the cell cycle, ATP-dependent chromatin remodeling, and JAK-STAT signaling pathways were also found to be overrepresented in this analysis. These pathways are known to be significantly disrupted during HPV infection (S4 Fig). These analyses indicate that A3B regulates the formation of specific sets of R-loops associated with TSS or TTS of genes critical for HPV pathogenesis.

### A subset of R-loops induced by APOBEC3B in HPV positive cells regulate cellular gene expression and DNA damage

**Fig 5:**
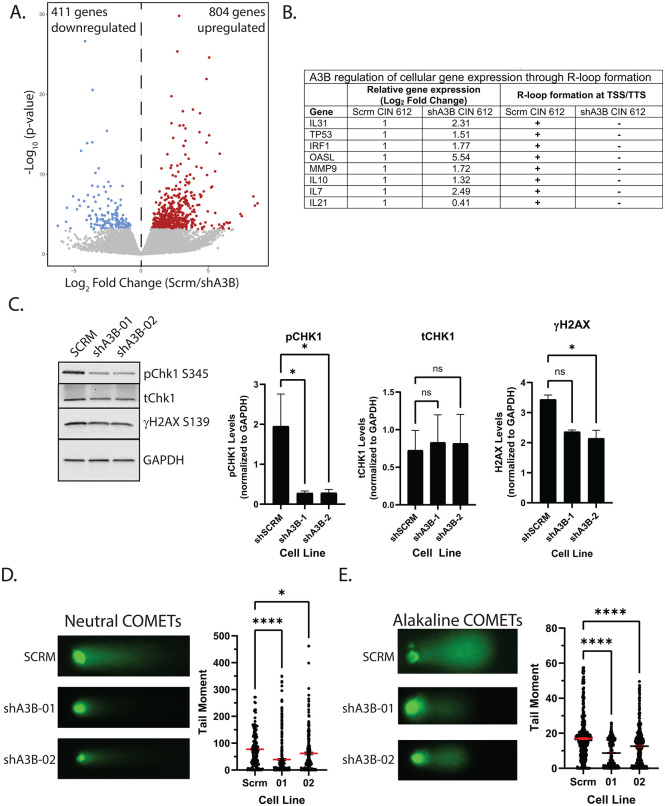
APOBEC3B induces DNA damage and regulates the expression of cellular immune genes in HPV positive cells. **(A)** Volcano plot of differentially expressed genes in scramble control or A3B depleted CIN 612 cells. Red dots represent upregulated and blue dots downregulated genes in A3B depleted CIN 612 cells compared to the scramble control. **(B)** A representative list of upregulated immune genes that no longer contained R-loops at their TSS/TTS in A3B depleted CIN 612 cells compared to the scramble control. **(C)** Western blot analysis of scramble control and A3B depleted CIN 612 cells for DNA damage repair factor, together with quantification of pChk1, tChk1, and γH2AX levels from three biological replicates (right). **(D)** Neutral and (E) alkaline COMET assays measuring dsDNA and ssDNA breaks, respectively, in scramble control and A3B depleted CIN 612 cells. Representative COMETs are displayed.

Cellular genomes contain large numbers of stable R-loops, but only a subset is involved in regulating gene expression. The above analyses identified R-loops that were dependent on A3B expression, and it was next critical to determine which of these are linked to altered gene expression. RNA-sequencing was performed on scramble control and A3B-depleted CIN 612 cells, and over 1,200 genes were found to be differentially expressed. Of these, approximately 800 were upregulated and more than 400 were downregulated in A3B-depleted cells compared to the scramble control (Fig 5A). Gene ontology analysis of the upregulated genes identified numerous pathways involved, including those related to immune signaling (response to interleukin-7, negative regulation of cAMP-mediated signaling) and chromatin remodeling (negative regulation of sister chromatid segregation, negative regulation of mitotic nuclear division, spindle checkpoint signaling) (S5A Fig). These pathways comprise genes that have lost R-loops located at TSS or TTS in A3B-depleted cells, including p53, IRF1, OASL, IL-7, and IL-10. The expression of these genes is increased in A3B depleted CIN 612 cells by over 2 fold (Fig 5B). In contrast, genes involved in keratinization, keratinocyte differentiation, and skin development were downregulated (S5B Fig). A representative list of 10 genes where R-loops are lost and whose expression is upregulated by A3B depletion, along with the associated level of change, is shown in Fig 5B.

As A3B can induce DNA damage through cytosine deamination and C-to-T transitions [62,63], it was important to investigate whether A3B depletion corresponded with reduced DNA breaks in HPV positive cells. Neutral COMET assays, which measure double-strand DNA breaks, were performed, and a ~ 50% reduction was observed in cells in which A3B was knocked down (Fig 5C). Furthermore, A3B is involved in the base excision repair (BER) pathway, so alkaline COMET assays were also conducted to compare relative levels of ssDNA breaks. A3B depletion resulted in a reduction of between 20–40% in ssDNA breaks (Fig 5D). Western analysis of DNA damage response factors revealed that pChk1 and γH2AX S139 levels were significantly reduced by ~2–3-fold (Fig 5E). These data support a role for A3B in inducing DNA breaks linked to R-loop formation, which can regulate viral and cellular gene expression in HPV positive cells.

## Discussion

The levels of APOBEC3B are elevated in both HPV positive cervical carcinomas and in cells that stably maintain viral episomes, serving as positive regulators of viral replication and expression. This is in contrast to what is observed in infections caused by other viruses, such as HIV, EBV, and HSV, where APOBEC proteins function as restriction factors [38,41,44,52].

In HSV or CMV infection, A3B is relocalized to the cytoplasm, whereas in HPV positive cells, they remain nuclear and bind to both viral as well as cellular sequences [50,51]. Insights into how A3B may function in HPV positive cells are provided by recent studies showing that A3B binds and regulates R-loop formation [46,47]*.* Importantly, the cytosine deaminase activity has been shown to be required for the resolution of RNA:DNA hybrids, while maintaining A3B DNA binding activity, is not sufficient to resolve these structures [46]. Previous studies have shown that R-loop levels in high-risk HPV positive cells are elevated due to the action of E6, acting through a combination of factors, including the inhibition of p53 [13] and similar activities that regulate A3B [34]. Importantly, A3B was found to bind to sequences at the HPV URR along with early polyA, which are the sites at which R-loops form, but less so on other viral coding regions.

Interestingly, in HPV positive cervical cancers and oropharyngeal cancers, which contain full-length genomes, A3B-induced mutations are found distributed throughout the viral genome, with potential hotspots near the URR and early polyA [64,65]. This may link the stable association of A3B with R-loops and the transient associations that induce mutations. Importantly, both A3B and R-loops are crucial for HPV replication and gene expression, supporting that these two factors are interlinked.

Given the linkage of A3B with R-loops, it was important to determine if R-loop formation was necessary for A3B binding or if A3B regulated R-loop formation. Overexpression of the R-loop-specific RNase, RNase H1, has been shown to reduce R-loop formation and, at the same time, impair HPV replication as well as expression [13,66,67]. In the present study, the overexpression of RNase H1 reduced R-loop levels on the HPV genome, accompanied by a corresponding 4-fold reduction in A3B binding at the URR and a 2-fold lower association at the early polyadenylation site in the HPV 31 genome. The ability of A3B to interact with cellular R-loop forming sequences was similarly tested by RNase H1 overexpression, and significant reductions were seen on cellular alleles that contained high R-loop levels in HPV positive cells (MYADM and RPL13a). Importantly, these reductions were about 2-fold, indicating that A3B binding was not completely ameliorated despite substantial reductions in R-loop formation. This suggests there are additional ways in which A3B can associate with DNAs. For instance, many R-loop-forming sequences are GC-rich and often result in the formation of G-quadruplexes that may stabilize ssDNA templates, which could retain A3B binding in the absence of R-loop formation [68], and this is an area for future study. It is clear, however, that the increased levels of R-loops present in HPV positive cells mediate a substantial portion of A3B’s interaction with cellular chromatin. Importantly, R-loops levels remain elevated throughout progression to cervical carcinomas and can provide sites for stable A3B binding, which could increase opportunities for A3B mutagenic activity at both viral and cellular sites.

Studies have implicated A3B as capable of either increasing the formation of R-loops or alternatively enhancing the resolution of R-loops [46,47]. Our studies indicate that in HPV positive cells, both scenarios occur depending on the gene. In HPV positive cells, R-loop levels are increased by more than 10-fold compared to those of normal keratinocytes [13,16], and A3B depletion reduced global R-loop levels by ~50%. This included reductions specifically in R-loop formation on the URR of the HPV 31 genome, but interestingly, not at the early poly (A) site. Multiple cellular factors regulate R-loop formation, including R-loop specific enzymes, like the helicase senataxin, and these factors may be dominant over the effects of A3B at specific loci. Our DRIP-seq analyses show that A3B also regulates the formation of R-loops on some, but not all, cellular loci, including genes critical for HPV pathogenesis, such as ATR, EP400, and RAD51 [59,61,69]. A3B was found to regulate the formation of R-loops preferentially at transcription start sites as well as termination sequences, but less so at intragenic regions. Previous studies correlated R-loop formation in HPV positive cells with the expression of genes controlling immune and DNA metabolizing pathways [16]. Consistent with these studies, reduced R-loop levels resulting from A3B depletion led to increased expression of immune genes, including IRF1, OASL, IL-7, and IL-10. A3B knockdown was also found to regulate gene expression in pathways such as endocytosis and HPV infection, including DNA damage repair factors, PI3K, and AKT signaling, as well as other metabolic pathways. Another intriguing finding is the association between A3B and the expression of genes involved in keratinocyte differentiation. While our studies focus on the involvement and importance of A3B in maintaining viral episomes and gene expression in undifferentiated keratinocytes, investigating its role upon differentiation is an important area for future studies. While the depletion of A3B impairs the transcript levels of E6 and E7, sufficient levels remain to support cell growth during maintenance of high-risk HPV episomes.

Our studies demonstrate that high levels of A3B were responsible for approximately 50% of DNA breaks in HPV positive cells, likely due to association with R-loops. This primarily consisted of double-strand breaks, with a moderate contribution from single-strand DNA breaks (~20–40%). Decreased levels of ATR transcription were observed, accompanied by a reduction in DNA damage markers, such as γH2AX and pChk1. Furthermore, A3B depletion results in a significant increase in p53 expression, which has previously been shown to regulate R-loop levels in HPV positive cells but not in normal cells. The increased levels of DNA breaks could also be attributed in part to several other mechanisms. For instance, A3B binding to R-loop forming sequences may slow down the resolution process, either by competing with other R-loop-resolving enzymes for binding templates or by resolving R-loops more slowly than enzymes like RNase H1. These stable R-loops would then serve as significant sources of replication transcription conflicts, which could collapse into double-stranded breaks. Second, A3B’s deaminase activity could result in downstream activation of the base excision repair (BER) pathway [62]. The resultant uracil moieties from deamination by A3B would require uracil DNA glycosylases to remove the uracil, leaving an abasic site. Additional processing of abasic sites then occurs through the downstream recruitment of DNA repair factors in the BER pathway; otherwise, these sites could stall or collapse replication forks. Previous studies have demonstrated that dsDNA break repair is impaired in HPV positive cells [14]; however, less is known about the role of ssDNA break repair pathways, such as BER, in the activation of R-loops, as well as A3B, which should be examined in the future.

The question remains as to how A3B can act as a positive regulator of HPV replication and expression, while in other viral systems it acts as a restriction factor. It is possible that A3B acts directly, in that it binds to and induces the formation of R-loops across the URR, which can act to promote viral gene expression. The resulting decreases in viral gene expression could result in the induced expression of innate immune genes sufficient to impair viral processes. Alternatively, the observation that A3B binding is partially dependent on R-loop formation across the cellular chromatin suggests it could also be regulating the expression of cellular genes detrimental to viral processes. As A3B has single strand DNA binding abilities along with its cytidine deaminase activity, R-loops may be stabilized at these cellular loci. Single-strand DNA-binding proteins have been shown to stabilize and increase the levels of R-loops when interacting with the displaced DNA strand, which is consistent with A3B’s activities in HPV positive cells [70].

Whether the cytidine deaminase activity or some other function of A3B has a role in positively regulating HPV pathogenesis is an important area for future study. A3B’s DNA binding activity may be the primary function critical for effects on R-loops, or perhaps it encodes a novel function that has previously been uncharacterized. Another possibility is that additional proteins are present in HPV positive cells that associate with or modify A3B, resulting in its proviral activities in HPV pathogenesis. These studies identifying the critical activities of A3B in HPV pathogenesis will require a detailed mutational analysis. Overall, our studies have shown that A3B plays a crucial proviral role in HPV pathogenesis, which contrasts with A3B’s restrictive role in regulating other DNA viruses. By inducing DNA damage and R-loop formation, A3B regulates the expression of both viral and cellular genes and promotes HPV replication.

## Materials and methods

### Tissue culture

Human foreskin keratinocytes were isolated from de-identified tissue samples obtained from the Skin Disease and Research Core at Northwestern University, as previously described. The primary foreskin keratinocytes and CIN612 9E cells, a clonal cell line derived from a cervical biopsy that stably maintains episomes of HPV31, were co-cultured with mitomycin treated 3T3 J2 fibroblasts in E media [71]. To generate cell lines expressing individual oncoproteins, primary cells were infected with recombinant viruses to stably express E6, E7, and E6 and E7, as previously described [72]. The J2 fibroblasts and HEK293T cells were cultured in DMEM containing penicillin/streptomycin and fetal bovine serum.

### Generation of A3B knockdown cell lines

Plasmids expressing shRNA sequences for APOBEC3B were obtained from Sigma Aldrich (S1 Table). These plasmid constructs were designed and purified for transduction as previously described [73]. The ampicillin-resistant E. coli glycerol stocks expressing shRNAs targeting A3B were grown up in Terrific broth (Fisher) with ampicillin at 100 μg/ml. The plasmids were purified with the EndoFree Maxi Plasmid Kit (Qiagen). Lentiviruses were generated by transfecting plasmids expressing shRNAs targeting A3B into HEK293 cells using Lipofectamine 2000 along with lentiviral helper plasmids (packaging and envelope, Addgene 2^nd^ Generation), and then the CIN612 cells were transduced. Cells were selected with puromycin (2μg/ml). The APOBEC3B knockdowns were validated by western blot and quantified using densitometry in ImageJ (NIH).

### Western blot analysis

Western blot analysis was performed as previously described [74]. Briefly, confluent 10 cm plates were harvested after removing the mitomycin treated J2 feeder cells by incubation in phosphate buffered saline with EDTA for 3–5 minutes. The cells were harvested and pelleted, following which the pellets were suspended in RIPA buffer with 10% SDS and lysed on ice for 20 minutes. The DNA was then sheared using a Bioruptor (Diagenode) under high power settings for 30 seconds on and 90 seconds off, for a total of 20 minutes. Laemmli buffer was added at a 1:4 ratio and heated at 95 °C for 5 minutes. These cell lysates were electrophoresed in 4–20% Criterion TGX precast midi protein gels (Bio-Rad) and then transferred to nitrocellulose membrane. The membranes were blocked in 5% bovine serum albumin (BSA), followed by incubation with primary antibodies at 4°C. The antibodies used in this study are listed in S2 Table. The membrane was then washed with ECL (Fisher), and images were captured using an Odyssey Fc Li-Cor (Li-Cor BioSciences) and quantified with ImageJ.

### Southern blot analysis

Whole cell nucleic acid extracts were performed on 10 cm dishes using Phenol/Chloroform. 5μg of DNA per sample was restriction enzyme digested with Xho1 for 1 hr at 37 °C, before being resolved at 40V for 18hr on a 1% agarose gel. DNA was depurinated and denatured by washing twice with 2 M HCl for 15 minutes and twice with 1.5 M NaOH for 45 minutes, respectively. DNA was then transferred to a positively charged (Zetaprobe) membrane via capillary action overnight. Radiolabeled HPV 31 genomic probe was generated using ^32^P-dCTP and the Random Primers DNA Labeling System (ThermoFisher). Membranes were equilibrated to hybridization buffer and blocked with salmon sperm DNA for 2.5 hr at 42 °C before addition of the denatured probe overnight at 42 °C. The following day, nonspecific binding of the radiolabeled probe was removed through a series of increasingly stringent washes with decreasing sodium citrate and increasing SDS. Membranes were then exposed to Hyperfilm (Cytiva) for 1, 3, and 7 days before being developed.

### Immunofluorescence

Immunofluorescence from tissue culture was carried out as previously described [13]. Cells were first fixed onto four-chamber slides (MatTek) with 4% paraformaldehyde for 15 minutes on ice. The antibodies used are listed above. Washes were performed with PBS before staining with DAPI and mounting. The slides were imaged through The Center for Advanced Microscopy at Northwestern on a Ti2 Nikon instrument.

### COMET assays

The COMET assays utilized the COMET Assay kit (Thermo Fisher), and the manufacturer’s published directions were followed for both neutral and alkaline COMETS. The cell-containing agar was dried on the slide for 30 min at 4°C before being transferred into the lysis buffer overnight. For the neutral COMET Assay, the slides were then placed 1X neutral running buffer for 30 min. These slides were then placed in the Trevigen COMETAssay ESII with chilled neutral running buffer and run at room temperature at 21V for 45 min. For the alkaline COMET Assay, the slides were removed from the lysis buffer and placed into alkaline unwinding solution for 20 min at 4°C in the dark. These slides were then transferred to the Trevigen COMETAssay ESII with chilled alkaline running buffer and run at room temperature at 21V for 30 min. For all COMET Assays, after electrophoresis, the slides were immersed twice in DI water for 5 min, then 70% ethanol for 5 min. The slides were dried at 37°C for 15 min. The slides were treated with 100 μL/circle of SYBR Gold for 30 min at room temperature and then imaged at The Center for Advanced Microscopy at Northwestern on a Ti2 Nikon instrument.

### RNA-seq analysis

Confluent 10 cm plates were treated with Versene to remove feeder cells before being harvested, and RNA was extracted from the cell pellets using Trizol extraction as previously described [5]. The samples were sequenced by the NUSeq Core. The single-stranded sequences were uploaded to Galaxy EU for bioinformatic analyses, such as DESeq2, and pathway analysis was completed through ShinyGo. Briefly, a quality check of the RNA-Seq reads was done with FASTQC (Galaxy Version 0.74 + galaxy1). Adaptors and low-quality bases were removed with Trimmomatic (Galaxy Version 0.39 + galaxy2). BAM files were created from the FATSQ files with RNA STAR (Galaxy Version 2.7.11a+galaxy1). Duplicates of mapping were marked by Picard Collect Sequencing Artifact Metrics (Galaxy Version 3.1.1.0). The alignments were assembled into potential transcripts with StringTie (Galaxy Version 2.2.3 + galaxy0). These transcripts were used to obtain feature counts with FeatureCounts (2.0.6 + galaxy0), and differential expression was determined from these counts by DESeq2 (2.11.40.8 + galaxy0). For mapping correlation between replicates and groups, Multi-BAM summary creation was managed with mamba (v23.1.0) and contained Python (v3.10). The primary analysis tool was deepTools (v3.5.5), which was used for coverage track generation with bamCoverage. Supporting packages were installed from the Bioconda and conda-forge channels, including pysam (v0.22.1) for BAM file handling, htslib (v1.20) and samtools (v1.20) for efficient sequence data processing, libdeflate (v1.20) for compression support, and NumPy (v1.26.0) to support scientific computing. Raw FastQ and DeSeq2 tabular data are publicly available in the NCBI GEO database (Accession number GSE324703).

### ChIP-qPCR

Confluent 10 cm dishes were fixed with 1% formaldehyde for 10 min, then quenched by adding glycine for 5 min. Samples were then processed as previously described [16]. Following immunoprecipitation and nucleic acid purification, primers were used in qPCR analyses to assess binding to various elements of viral and cellular chromatin. Primer sets are listed in S3 Table.

### DRIP-qPCR

DRIP-qPCR analysis was performed as previously described. Briefly, DNA extracted via Phenol/Chloroform was sonicated with a Bioruptor for 20 minutes at high output with cycles of 30s on/90s off or treated with 1U of mung-bean nuclease for 1 hr. RNase H treatment was performed on a subset of samples by treating with 5U RNase H for 2–3 hr at 37 °C. Precleared magnetic protein G beads (Dynabeads, Fisher) with 2μg of S9.6 were added to 25 – 50μg of fragmented DNA in ChIP collection buffer. Complexes were allowed to form overnight at 4 °C while rotating. The following day, samples were washed nine times with RIPA buffer for 5 min per wash and equilibrated in TE for 5 minutes before immunoprecipitated sequences were eluted using 10mM Tris, 2mM EDTA, 10% SDS for 30 min at 65 °C. Samples were resuspended in diH2O, and qPCR analysis was performed using primers previously used for R-loop analyses [16].

### DRIP-sequencing

DRIP analysis was performed as described above for DRIP-qPCR. Instead of performing qPCR analysis, samples were shipped to Admera Biosciences (NJ) for DRIP-sequencing. Mapped BAM files were provided by Admera Biosciences, and downstream bioinformatic analyses were performed using the open-source platform, Galaxy US/EU. The bioinformatic pipeline was the same as previously described for ChIP-seq [16]. Raw FastQ, mapped BAM, and MACS2 peak-calling files are publicly available in the NCBI GEO database (Accession No. GSE324516).

### S9.6 dot blot assay

The dot blot protocol for quantifying global R-loops was performed as previously described [13]. Briefly, 1.2μg of extracted DNA from cell lysates by Phenol/Chloroform was spotted onto a positively charged membrane (Zeta-probe) before being stained with methylene blue to normalize nucleic acid content. Methylene blue was removed by washing with 100% EtOH for 3 min, and the membranes were blocked with 5% BSA in TBST for 1 hr. The membrane was probed overnight at 4°C with the S9.6 antibody (1:4,000). The membrane was then washed before being incubated with the secondary antibody. Blots were developed in ECL (Fisher, 4500085) and imaged using Odyssey FC LiCor (LiCor BioSciences).

### Statistical analysis and software usage

Statistical analysis and figure generation were performed using GraphPad Prism 10. Unless otherwise noted, three replicates were performed for each experiment, with either a representative image shown or depicted by a graph. P-values are as follows: p < 0.05, *; p < 0.01, **; p < 0.001, ***; and p < 0.0001, ****. Error bars are plotted as the standard error of the mean. Figures were compiled with Adobe Suite. The Galaxy EU/US open-source platform was used for DRIP-sequencing and RNA-sequencing analysis. COMET scoring was done in COMETScore. Immunofluorescence was performed using Nikon NIS-Elements AR software.

## Supporting information

S1 Fig APOBEC3B levels detected with a commercial antibody and analysis of A3B depletion on CIN 612 cell growth. (A) Western blot analysis comparing the commercially available A3B antibody (Cell Signaling, 5210-87-13) and the previously published antibody, primarily used in this study [48]. Scramble control and A3B depleted CIN 612 cells were used for this analysis. (B) Growth curves of scramble control and A3B depleted CIN 612 cells over the course of 3 days.
(EPS)

S2 Fig APOBEC3B is upregulated and important for viral gene expression in primary keratinocytes stably maintaining HPV 16 and 18 episomes. (A) Western blot analysis comparing APOBEC3B levels between HFK, HFK 16, HFK 16, HFK 31 and CIN 612 cells (n = 3, a representative image is shown). (B) APOBEC3B RNA and protein levels were significantly depleted upon lentiviral delivery of shRNAs targeting APOBEC3B in HFK 16 and 18 cells. (C) Depletion of APOBEC3B reduces E6 and E7 expression in HFK 16 cells (left) and HFK 18 cells (right) (n = 3; **, p < 0.01; ****, p < 0.0001).
(EPS)

S3 Fig RNase H1 overexpression in CIN 612 cells preferentially removes R-loops on cellular genes with elevated R-loop levels. (A,B) DRIP-qPCR on scramble control and RNase H1 overexpressing CIN 612 cells, analyzing cellular genes prone to R-loop formation, known to either contain elevated R-loop levels (MYADM & RPL13a) or similar levels (EGR1 & SNRPN) in HPV positive cells compared to normal cells. (C) A3B RNA levels in HFK, CIN 612, or RNase H1 overexpressing CIN 612 cells from RNA sequencing analysis previously published [16].
(EPS)

S4 Fig KEGG pathways and representative genes with reduced R-loop formation upon A3B depletion in CIN 612 cells. (EPS)

S5 Fig Gene ontology analysis of (A) upregulated and (B) downregulated genes upon A3B depletion in CIN 612 cells. (EPS)

S1 Table XXX shRNA sequences used to target A3B. (DOCX)

S2 Table Antibodies used in this study. (DOCX)

S3 Table Primer sets used in this study. (DOCX)
