# Supplementary material for: APOBEC3B regulates HPV replication by inducing R-loop formation and DNA damage
Source: PLoS Pathog. 2026 Mar 23;22(3):e1014088. doi: 10.1371/journal.ppat.1014088 (PMC13035229; doi:10.1371/journal.ppat.1014088)
Supplement: S1 Table — (DOCX) [file ppat.1014088.s006.docx]

| S1 Table - shRNA sequences used in this study | | |
| --- | --- | --- |
| **shRNA cell line** | **Sequence** | **Targeting Location** |
| shA3B-01 | Previously published sequence | Burns et al, 2013 |
| shA3B-02 | 5’-GCAAAGCAATGTGCTCCTGAT-3’ | 3’ UTR |
| shA3B-03 | 5’-GCACGCTAAAGGAGATTCTCA-3’ | CDS |
| shA3B-04 | 5’-CCTTGGTACAAATTCGATGAA-3’ | CDS |
