# Supplementary material for: APOBEC3B regulates HPV replication by inducing R-loop formation and DNA damage
Source: PLoS Pathog. 2026 Mar 23;22(3):e1014088. doi: 10.1371/journal.ppat.1014088 (PMC13035229; doi:10.1371/journal.ppat.1014088)
Supplement: S2 Table — (DOCX) [file ppat.1014088.s007.docx]

| S2 Table – Antibodies used in this study | | | | |
| --- | --- | --- | --- | --- |
| **Antibody** | **Host Animal** | **Dilution for immunofluorescence** | **Dilution for Western blot** | **Distributor, cat. No.** |
| DNA-RNA Hybrid (S9.6) | Mouse, monoclonal |  | 1:1000 | Millipore Sigma, MABE1095 |
| GAPDH | Mouse, monoclonal |  | 1:4000 | Santa Cruz Biotechnology, sc47724 |
| H2AX (ser139) | Rabbit, monoclonal |  | 1:4000 | Cell Signaling Technologies, 9718S |
| Rig-I | Rabbit, monoclonal |  | 1:1000 | Cell Signaling Technologies, 3743S |
| Anti-Rabbit IgG, HRP-linked |  |  | 1:4000 | Cell Signaling Technologies, 7074 |
| Anti-Mouse IgG, HRP-linked |  |  | 1:4000 | Cell Signaling Technologies, 7076 |
| SERPINB3 Monoclonal Antibody (OTI1G9), TrueMAB™ 30 ul | Mouse, monoclonal |  | 1:1000 | Thermo Fisher, TA506890S |
| Chk1 (2G1D5 | Mouse, monoclonal |  | 1:1000 | Cell Signaling Technologies, 2360S |
| APOBEC3B | Rabbit | 1:100 | 1:1000 | A generous gift of the D'Aquila Lab |
| Phospho-CHK1 (Ser345) | Rabbit, monoclonal |  | 1:500 | Thermo Fisher, MA5-15145 |
| Phospho-ATR (Ser428) | Rabbit, polyclonal |  | 1:500 | Cell Signaling, 2853 |
| ATR (2B5) | Mouse, monoclonal |  | 1:100 | Thermo Fisher, MA1-23158 |
| Phospho-ATM (Ser1981) | Rabbit, monoclonal |  | 1:500 | Cell Signaling, 13050 |
| ATM | Rabbit, polyclonal |  | 1:500 | Fisher Scientific Novus Biologicals, NB100-104 |
| beta Tubulin | Rabbit, polyclonal |  | 1:4000 |  |
| Anti-RPA32/RPA2 antibody [9H8] | Mouse, monoclonal |  | 1:1000 | abcam, ab2175 |
| Chk2 (1C12) | Mouse, monoclonal |  | 1:1000 | Cell Signaling, 3440s |
| Phospho-Chk2 (Thr68) | Rabbit, monoclonal |  | 1:500 | Cell signalling, 2197S |
