# Supplementary material for: APOBEC3B regulates HPV replication by inducing R-loop formation and DNA damage
Source: PLoS Pathog. 2026 Mar 23;22(3):e1014088. doi: 10.1371/journal.ppat.1014088 (PMC13035229; doi:10.1371/journal.ppat.1014088)
Supplement: S3 Table — (DOCX) [file ppat.1014088.s008.docx]

| S3 Table – Primer sets used in this study | | |
| --- | --- | --- |
| **Oligonucleotides** |  |  |
| Primer Sets | Forward | Reverse |
| **Cellular Regions** | - | - |
| GAPDH | 5 ′ CTC TGA TTT GGT CGT ATT GG 3 ′ | 5 ′ GTA AAC CAT GTA GTT GAG GTC 3 ' |
| APOBEC3B | 5 ' CCC GGA CTG TGT GTG 3 ' | 5 ' GCG GTG CAG GAA TGC 3 ' |
| APOBEC3B-RH | 5 ' GAC CCT TTG GTC CTT CGA C 3 ' | 5 ' GCA CAG CCC CAG GAG AAG 3 ' |
| MYADM | 5 ′ CGT AGG TGC CCT AGT TGG GAG 3 ′ | 5 ′ TCC ATT CTC ATT CCC AAA CC 3 ′ |
| RPL13a | 5 ′ AAT GTG GCA TTT CCT TCT CG 3 ′ | 5 ′ CCA ATT CGG CCA AGA CTC TA 3 ′ |
| EGR1 | 5 ′ GAA CGT TCA GCC TCG TTC TC 3 ′ | 5 ′ GGA AGG TGG AAG GAA ACA CA 3 ′ |
| SLC35B2 | 5 ′ AAG TCT TGC CCT AGC TGT GCT 3 ′ | 5 ′ GCC TAC ACC GCT TGT GCT TTT 3 ′ |
| SNRPN | 5 ′ GCC AAA TGA GTG AGGATG GT 3 ′ | 5 ′ TCC TCT CTG CCT GAC TCC AT 3 ' |
| ALU elements | 5 ′ ACG AGG TCA GGA GAT CGA GA 3 ′ | 5 ′ CTC AGC CTC CCA AGT AGC TG 3 ' |
| **HPV31 Elements** | - | - |
| Early PolyA | 5 ′ GGT ATT GGT ATT GGT ATT GG 3 ' | 5 ′ ACC CAT ACT ACC ATA CCT TA 3 ′ |
| Upstream regulatory region (URR) | 5 ′ GAT GCA GTA GTT CTG CGG TTT 3 ' | 5 ′ TAT GTT GGC AAG GTG TGT TAG G 3 ' |
| E6 | 5 ′ GAC CTC GGA AAT TGC 3 ′ | 5 ′ AAC ATG CTA TGC AAC GTC CTG 3' |
| E7 | 5 ′ AAT TAC CCG ACA GCT CAG ATG 3 | 5 ′ GGC ACA CGA TTC CAA ATG AG 3' |
| E1 | 5 ′ GAC AGA CAG ACA GGG G 3 ′ | 5 ′ CCC GCT GTC TGG AAG TTC 3 ′ |
| **HPV16 Elements** | - | - |
| Early PolyA | 5 'AGG ATA CTT CGT TGC TGC 3 ' | 5 ' TGT TCA TGA AGG AAT ACG 3 ' |
| Upstream regulatory region (URR) | 5 ' AGG CTC TGG GTC TAC TGC 3 ' | 5 ' AAA CTG ATC TAG GTC TGC 3 ' |
| E6 | 5 ' TCT ACA ACT GCT AAA CGC 3 ' | 5 ' GCA GGT CAG GAA AAC AGG 3' |
| E7 | 5 ' AAT CAC TAT GCG CCA ACG 3 ' | 5 ' ATC GGT TTG CAC ACA CCC 3 ' |
| **HPV18 Elements** | - | - |
| Early PolyA | 5 ' GCC CAT GTT ACT ATT GCA 3 ' | 5 ' GGT GGG ATA CCA TAC TTT 3 ' |
| Upstream regulatory region (URR) | 5 ' GGC GCG CCT CTT TGG CGC 3 ' | 5 ' CAC CGT TTT CGG TCC CGA 3 ' |
| E6 | 5 ' CAC GGC GAC CCT ACA AGC 3 ' | 5 ' CTC TGC GTC GTT GGT GAC G 3 ' |
| E7 | 5 ' GGT TGC ATG CTT TTT GGC 3 ' | 5 ' ACG TTT TGT GCG TTT TGC 3 ' |
